# Belinostat, a potent HDACi, exerts antileukaemic effect in human acute promyelocytic leukaemia cells *via* chromatin remodelling

**DOI:** 10.1111/jcmm.12550

**Published:** 2015-04-11

**Authors:** Giedre Valiuliene, Ieva Stirblyte, Dovile Cicenaite, Algirdas Kaupinis, Mindaugas Valius, Ruta Navakauskiene

**Affiliations:** a Department of Molecular Cell Biology, Institute of Biochemistry, Vilnius UniversityVilnius, Lithuania; b Proteomics Centre, Institute of Biochemistry, Vilnius UniversityVilnius, Lithuania

**Keywords:** APL, HDACi, belinostat, epigenetics, granulocytic differentiation

## Abstract

Epigenetic changes play a significant role in leukaemia pathogenesis, therefore histone deacetylases (HDACis) are widely accepted as an attractive strategy for acute promyelocytic leukaemia (APL) treatment. Belinostat (Bel, PXD101), a hydroxamate-type HDACi, has proved to be a promising cure in clinical trials for solid tumours and haematological malignancies. However, insight into molecular effects of Bel on APL, is still lacking. In this study, we investigated the effect of Bel alone and in combination with differentiation inducer retinoic acid (RA) on human promyelocytic leukaemia NB4 and HL-60 cells. We found that treatment with Bel, depending on the dosage used, inhibits cell proliferation, whereas in combination with RA enhances and accelerates granulocytic leukaemia cell differentiation. We also evaluated the effect of used treatments with Bel and RA on certain epigenetic modifiers (HDAC1, HDAC2, PCAF) as well as cell cycle regulators (p27) gene expression and protein level modulation. We showed that Bel in combination with RA up-regulates basal histone H4 hyperacetylation level more strongly compared to Bel or RA alone. Furthermore, chromatin immunoprecipitation assay indicated that Bel induces the accumulation of hyperacetylated histone H4 at the p27 promoter region. Mass spectrometry analysis revealed that in control NB4 cells, hyperacetylated histone H4 is mainly found in association with proteins involved in DNA replication and transcription, whereas after Bel treatment it is found with proteins implicated in pro-apoptotic processes, in defence against oxidative stress and tumour suppression. Summarizing, our study provides some novel insights into the molecular mechanisms of HDACi Bel action on APL cells.

## Introduction

Acute promyelocytic leukaemia (APL) is an acute myeloid leukaemia (AML) subtype, characterized by block of granulocytic differentiation and accumulation of promyelocytes in the bone marrow and blood 1. Acute promyelocytic leukaemia patients possess specific reciprocal chromosomal translocations involving the retinoic acid receptor α (*RARA*) gene and one of its gene fusion partners. The pathogenesis of this disease in most cases is associated with the formation of chimeric PML-RARA protein (>98%) 2. It has been demonstrated that in APL, fusion proteins of the RARA recruit histone deacetylases (HDACs) containing co-repressor complexes 3, which in turn deacetylate and silence genes crucial for haematopoietic differentiation 4. Treatment with pharmacological doses of all-trans retinoic acid (RA) has been shown to force APL cells differentiation into mature granulocytes 5. However, resistance to the cytodifferentiating effects of RA is frequently acquired during drug therapy 6. Therefore, APL treatment requires other clinical approaches. Numerous investigations showed that there is a rationale to use RA in combination with epigenetic drugs such as HDAC inhibitors (HDACis) 7,8.

One of the most promising agents in this category—belinostat, which is a novel and potent hydroxamate-type HDACi, was shown to inhibit 1-st and 2-nd class HDACs enzymatic activity *in vitro* 9. Belinostat exerts its anti-deacetylase action *via* its hydroxamic acid moiety binding to zinc ion in enzymes’ catalytic domains and blocking substrate access 10. Previous studies have shown its activity resulting in cell cycle arrest, apoptosis and inhibition of cell proliferation 11,12. Belinostat has been already tested in phase I and II clinical trials against solid tumours, such as malignant pleural mesothelioma 13, thymic epithelial tumours 14, unresectable hepatocellular 15, ovarian, fallopian tube or primary peritoneal carcinoma 16,17. It should be emphasized that in solid tumours belinostat demonstrated more promising effects in combination with traditional chemotherapy, rather than applied as a single therapy 18. Belinostat also has been used in phase II trials as monotherapy in newly diagnosed AML 19. However, as a single agent it was shown to have minimal effect. In contrast, belinostat in combination with the proteasome inhibitor bortezomib elicited pro-apoptotic effect in AML and ALL cell lines and primary blasts, whereas analogous treatment was non-toxic to normal CD34(+) cells 20. In addition, belinostat in combination with decitabine, theophyline and RA has shown to exert anti-proliferative effect on AML blasts 21.

All this available data suggest that belinostat in combination with other drugs may be a valuable strategy for APL treatment. Therefore, a further more profound investigation is necessary to determine its applicability for APL differentiation therapy and to decipher belinostat’s molecular effects on APL cells.

In this study, we investigated the application of belinostat for leukaemia cell granulocytic differentiation using APL cell line NB4 (FAB-M3) and promyelocytes resembling HL-60 cells (FAB-M2), although not bearing typical APL translocation t(15;17). To unravel molecular mechanisms involved in belinostat’s action, we further examined its effect on APL cells gene and protein expression (HDAC1, HDAC2, PCAF, p27), as well as on histone H4 hyperacetylation level. Furthermore, we examined belinostat’s effect on composition modulation of protein complexes associated with hyperacetylated histone H4.

## Materials and methods

### Cell culture

The human APL cells NB4 and HL-60 (from DSMZ, GmbH, Braunschweig, Germany) were maintained in RPMI 1640 medium supplemented with 10% foetal bovine serum, 100 U/ml penicillin and 100 mg/ml streptomycin (Gibco, Grand Island, NY, USA) in a humidified incubator at 37°C with 5% CO_2_. For each experiment, logarithmically growing cells were seeded at a density of 0.5 × 10^6^ cells/ml in 5 ml of medium. According to previous publication 22 cells were exposed to 0.2 and 2.0 μM Belinostat (Selleck Chemicals, Houston, TX, USA) alone or in combination with 1 μM RA (Sigma-Aldrich, St. Louis, MO, USA). The agents were left in the cell media for the duration of the experiment.

### Assessment of granulocytic cell differentiation and cell cycle analysis

The degree of granulocytic differentiation was evaluated by cells ability to reduce soluble nitro blue tetrazolium (NBT) to insoluble blue-black formazan after stimulation with phorbolmyristate acetate. Nitro blue tetrazolium positive stained cells were counted in five consecutive non-overlapping microscopic fields at a magnification of 400. The average percent of NBT positive cells per high power field was calculated. Three independent experiments were performed and their results were averaged. Flow cytometric analysis of cell cycle distribution was performed as described earlier 22.

### RNA extraction, cDNA synthesis and RT-qPCR assay

All procedures were performed as indicated earlier 22. The primer sets for the tested genes are listed in the Table1.

**Table 1:** Primer sets of tested genes

Analysis type	Phenotypic end-points	Gene	Sequence	Product size (bp)	Tm (°C)	Resource
RT-qPCR	Epigenetic modifiers	HDAC1	F: CAAGCTCCACATCAGTCCTTCC	102	60	26
			R: TGCGGCAGCATTCTAAGGTT			
		HDAC2	F: AGTCAAGGAGGCGGCAAAA	103	60	26
			R: TGCGGATTCTATGAGGCTTCA			
		PCAF	F: GGCCGAGGAGTCTTGTAAAT	649	60	Primer Bank
			R: AGTGAAGACCGAGCGAAGCA			
	Cell cycle regulators	P27	F: TAATTGGGGCTCCGGCTAACT	116	60	Primer Bank
			R:TGCAGGTCGCTTCCTTATTCC			
	Reference gene	GAPDH	F: TCCATGACAACTTTGGTATCG	471	60	27
			R: TGTAGCCAAATTCGTTGTCA			
ChIP-qPCR	Cell cycle regulators	P27	F: GGCCTCCCCCGCAGACCAC	382	60	Self-designed based on Ref. 28
			R: GTTCCGCCACCTCCCCTCGTTCC			
	Transcription factors	C/EBPα	F: GTGCAGCCTCGGGATACTC	70	60	Self-designed based on Ref. 29
			R: CTCCTCCTGCCTGCCCTA			
		C/EBPε	F: GCTAACCGGAATATGCTAATCAG	296	60	Self designed based on Ref. 30
			R: CCTTTCAGAGACACCTGCTC			

### Total protein isolation and Western blot analysis

For total protein extraction control and treated cells (2 × 10^6^) were washed twice with PBS, incubated with Benzonase® Nuclease (Novagen, Merck KGaA, Darmstadt, Germany) 1 to 10 μl of pellet for 30 min. on ice, later resuspended in 10 volumes of 2× SDS protein loading buffer and heated for 5 min. in 95°C. Protein lysate then was centrifuged at 11,904 g. for 5 min. at 10°C and used for successive analysis. Five microlitre of protein specimens was run on gradient (7.5–15%) polyacrylamide gel. Electrophoresed proteins were transferred to ImmobilonTM PVDF transfer membrane (Millipore, Bedford, MA, USA). Immunoblotting was performed with antibodies against PCAF (Abcam, Cambridge, UK), HDAC1, HDAC2, GAPDH and p27 (Cell Signaling, Danvers, MA, USA). Immunoreactive bands were visualized with an enhanced chemiluminescence (WesternBrightTM ECL kit, Advansta Corporation, Menlo Park, CA, USA), according to the manufacturer’s instructions. Blots were scanned and optical density evaluated using ImageJ software (1.45s) (NIH, Bethesda, MD, USA).

### Evaluation of global DNA methylation

For global DNA methylation analysis control and treated NB4 cells (1 × 10^6^) were washed with PBS and genomic DNA extracted using ZR Genomic DNA™ – Tissue MiniPrep (Zymo Research, Irvine, CA, USA). Extracted genomic DNA was used for a subsequent 5-mC DNA quantity evaluation, using 5-mC DNA ELISA Kit (Zymo Research), according to the manufacturer’s instructions. Data were represented as a fold change compared with control.

### Chromatin immunoprecipitation for qPCR analysis

Chromatin immunoprecipitation (ChIP) assay was performed with a previously described method with specific modifications 23. For ChIP assay 5–10 mg of antibody to hyperacetylated histone H4 (Upstate Biotechnology, Lake Placide, NY, USA) was used per 15–20 mg DNA. qPCR analysis of immunoprecipitated DNA was performed with Maxima® SYBR Green qPCR Master Mix on the Rotor-Gene 6000 system. The primer sets for the tested genes are listed in the Table1. For data evaluation, the percent input was calculated, according to the formula: 100 · 2^(Adjusted input−Ct (IP)^.

### Chromatin immunoprecipitation for mass spectrometry analysis

All ChIP procedures were carried out as discussed in the previous section. Protein A/G PLUS-Agarose –Antibody–Protein complexes were denatured in 7 M Urea, 2 M Thiourea, 40 mM DTT solution, with continuous rotation at 50 r.p.m. in the temperature controlled shaker for 0.5 hrs at 20°C. Complexes were centrifuged (180 g, 7 min., 20°C) and extraction repeated additional three times. All four extracted fractions were combined and subjected to further mass spectrometry (MS) analysis.

### Mass spectrometry and data analysis

Extracted proteins were applied on Amicon Ultra-0.5 mL 30 kD centrifugal filter unit (Sigma-Aldrich). Trypsin digestion was carried out according to a modified Filter-aided sample preparation (FASP) protocol as described by Wisniewski *et al*. 24. Liquid chromatography separation of trypsin cleaved peptides and mass spectrometric analysis were performed as described earlier 25.

Raw data files were processed and searched using ProteinLynx Global SERVER (PLGS) version 2.5.2 (Waters Corporation, Manchester, UK). The following parameters were used to generate peak lists: (*i*) minimum intensity for precursors was set to 100 counts, (*ii*) minimum intensity for fragment ions was set to 30 counts, (*iii*) intensity was set to 500 counts. Processed data were analysed using trypsin as the cleavage protease, one missed cleavage was allowed and fixed modification was set to carbamidomethylation of cysteines, variable modification was set to oxidation of methionine. Minimum identification criteria included two fragment ions per peptide, five fragment ions per protein and minimum of two peptides per protein. The false discovery rate (FDR) for peptide and protein identification was determined based on the search of a reversed database, which was generated automatically using PLGS when global FDR was set to 4%. Functional protein association networks were constructed using STRING database (http://string-db.org/).

### Statistical analysis

Unless otherwise specified, all experiments were repeated at least three times. Data were expressed as mean values with SDs. For statistical analysis two-sample Student’s *t*-test was used.

## Results

### Effects of belinostat alone and in combined treatment with RA on NB4 and HL-60 cells growth and cell cycle arrest

**Figure 1:**
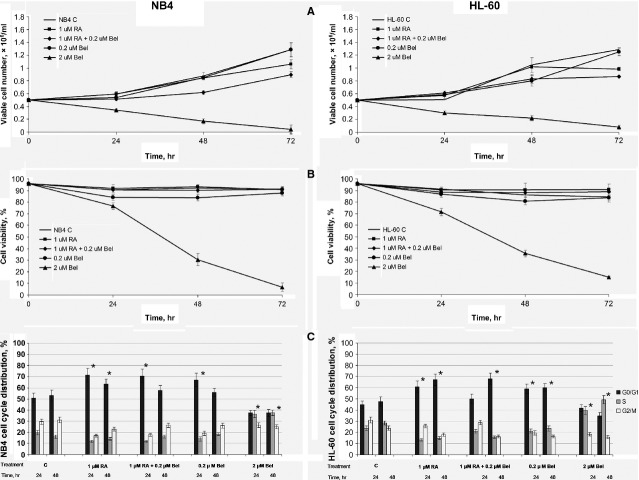
Effects of Bel alone and in combined treatment with RA on NB4 and HL-60 cells growth, viability and cell cycle arrest. Control and NB4, HL-60 cells treated with 0.2 μM, 2 μM Bel, 1 μM RA and with 0.2 μM Bel + 1 μM RA, were subjected to evaluation of cell growth (A), cell viability (B) and cell cycle distribution (C). At each time-point indicated, aliquots of the cultures were subjected to 0.2% trypan blue staining for cell growth (A) and viability (B) determination. Results are given as mean (± SD, *n* = 3). Statistically significant differences between treatment with RA alone and with combined treatment Bel + RA were observed in NB4 cells (upon 24 hrs treatment *P* < 0.05, upon 48 hrs treatment *P* < 0.01; A). The cell cycle phase distribution (%; C) was assayed flow cytometrically from the DNA frequency distribution histograms of PI stained cells. Results are given as mean (±SD, *n* = 3). *P < 0.01 (difference between untreated and treated samples).

We determined the impact of treatment with Bel (0.2 and 2 μM) alone and its combined treatment together with RA (0.2 μM Bel + 1 μM RA) on NB4 and HL-60 cells growth. We demonstrated (Fig.1A), that 0.2 μM Bel alone has no observable inhibitory effect neither on NB4 nor on HL-60 cells proliferation. However, 2 μM Bel suppressed both cell lines growth and down-regulated their viability tremendously (Fig.1B; *P* < 0.01). Noticeably, upon combined treatment with 0.2 μM Bel + 1 μM RA no loss in cell viability was detected (Fig.1B). However, combined treatment inhibited NB4 and HL-60 cells proliferation more efficiently compared with treatments with 0.2 μM Bel and 1 μM RA alone. Statistically significant differences between treatment with RA alone and with combined treatment Bel + RA were observed in NB4 cells (upon 24 hrs treatment *P* < 0.05, upon 48 hrs treatment *P* < 0.01). Cell cycle analysis (Fig.1C) showed that treatments with 0.2 μM Bel + 1 μM RA, 1 μM RA and, to a lesser extent, with 0.2 μM Bel, arrest NB4 and HL-60 cells in G_0_/G_1_ cell cycle stage (*P* < 0.01), whereas treatment with 2 μM Bel blocks cell cycle in S phase (*P* < 0.01).

### Belinostat enhances RA-induced NB4 and HL-60 cells granulocytic differentiation

**Figure 2:**
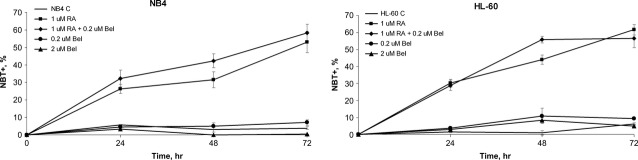
Effects of Bel, RA and their combined treatments on NB4 and HL-60 cells granulocytic differentiation. Control and NB4, HL-60 cells treated with 0.2 μM, 2 μM Bel, 1 μM RA and with 0.2 μM Bel + 1 μM RA at indicated time-points (24–72 hrs) were subjected to granulocytic differentiation analysis, using NBT+ test. Results are given as mean (± SD, *n* = 3).

For granulocytic differentiation evaluation cells were treated with 1 μM RA, 0.2 μM Bel alone and their combination (1 μM RA + 0.2 μM Bel). Nitro blue tetrazolium test revealed that Bel alone is not sufficient to induce NB4 and HL-60 cells differentiation. However, we showed that it enhances and accelerates RA-induced granulocytic differentiation (Fig.2), although not statistically significantly. The most pronounced effect was visible on NB4 cell line, therefore NB4 cells were chosen for subsequent gene and protein expression, as well as ChIP analysis.

### Belinostats effect on NB4 cells’ gene expression

**Figure 3:**
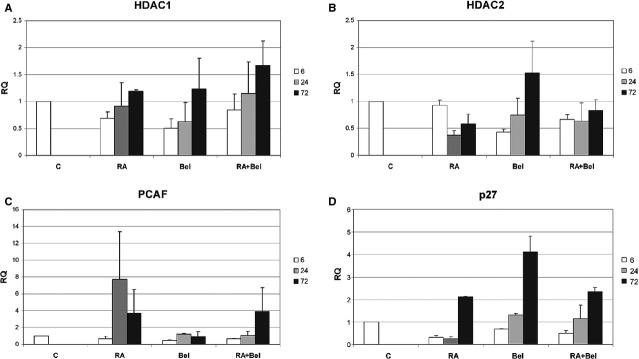
Effects of Bel, RA and their combined treatments on NB4 cells gene expression. (A–D) Control NB4 cells (C) and cells treated with 1 μM RA (RA), 0.2 μM Bel (Bel) and 1 μM RA + 0.2 μM Bel (RA + Bel) were subjected to RT-qPCR analysis. Target gene expression: HDAC1 (A), HDAC2 (B), PCAF (C) and p27 (D) was normalized to GAPDH reference gene. Fold change in gene expression (denoted as RQ – relative quantity) data is represented as mean (± SD, *n* = 3).

RT-qPCR analysis revealed the down-regulation of HDAC1 gene expression (Fig.3A) after treatment with 0.2 μM Bel, 1 μM RA and combined treatment 0.2 μM Bel + 1 μM RA. The strongest effect was induced by Bel alone, as Bel instantly decreased HDAC1 gene expression. Effect was still observable after 24 hrs incubation, whereas later the HDAC1 gene expression level has been restored. Consistently with this data, Bel alone was the most prominent in sudden HDAC2 gene expression reduction (Fig.3B). Although after longer incubation periods with Bel HDAC2 gene expression was restored, combined treatment Bel + RA restricted HDAC2 expression even after 72 hrs incubation.

We were interested, if Bel also has an impact on PCAF gene expression. From data presented in Figure3C it is evident, that Bel alone had no effect on PCAF gene expression, whereas RA dramatically up-regulated PCAF mRNA level. Only Combined treatment Bel + RA had reached RA effect after 72 hrs incubation. In contrast, Bel alone, and to a lesser extent in combination with RA, compared with RA alone, was more efficient in p27 gene expression induction (Fig.3D).

### Belinostat influence on NB4 cells’ protein level modulation

**Figure 4:**
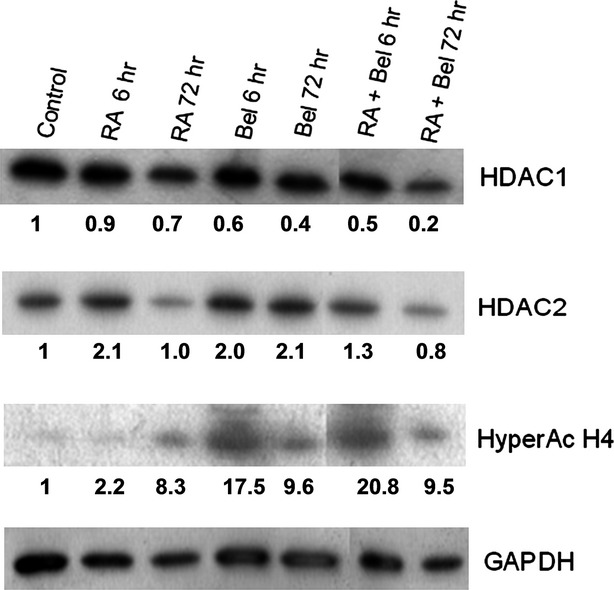
Effects of Bel, RA and their combined treatments on NB4 cells protein level regulation. NB4 cells were treated with 1 μM RA (RA), 0.2 μM Bel (Bel) and 1 μM RA + 0.2 μM Bel (RA + Bel) for 6–72 hrs. At indicated time-points total protein was isolated from control and treated cells. Identical amount of proteins were separated by SDS/PAGE electrophoresis in 7–15% acrylamide gradient gel, transferred onto PVDF membrane and subjected to western blot analysis, using antibodies against examined proteins (Hyper Ac H4, HDAC1, HDAC2) and GAPDH as a loading control. Blots were scanned and optical density evaluated using ImageJ software. The data are representative of three independent experiments showing similar results. The fold change values compared with control are indicated; SDs <15%.

To evaluate molecular mechanisms that Bel modulates in greater detail, we investigated the effect of 0.2 μM Bel treatment (alone or in combination with 1 μM RA) on histone H4 hyperacetylation level, as well as HDAC1 and HDAC2 protein level regulation (Fig.4).

The most efficient increase in NB4 cells’ histone H4 hyperacetylation level was observed after combined treatment 0.2 μM Bel + 1 μM RA. The effect was rapid (evident after 6 hrs incubation) and highly pronounced (histone H4 hyperacetylation level increased 21-fold compared with control cells). After a sudden initial increase, histone H4 hyperacetylation level later declined. However, after 72 hrs treatment with Bel + RA it was still approximately 10-fold greater compared with control cells. The similar effect was observable after 72 hrs treatment with RA or Bel alone.

Combined treatment of Bel + RA also had the most obvious effect on HDAC1 protein level down-regulation. After 6 hrs incubation with combination of these agents HDAC1 protein level has dropped more than twofold, after 72 hrs incubation HDAC1 protein level was reduced up to five times compared with untreated cells. From the presented data it is evident that RA alone is insufficient to reduce HDAC1 protein level, however, it is capable to enhance Bel effect on HDAC1 protein level down-regulation. In contrast to HDAC1, HDAC2 protein level was shown to be up-regulated immediately upon treatment with Bel, RA or their combination. Later HDAC2 was restored to its previous level (except after treatment with Bel).

### Combined treatment with belinostat and RA effects NB4 cells global DNA methylation

We evaluated the effect of treatment with RA and Bel, as single agents, and their combined treatment Bel + RA on NB4 cells’ global DNA methylation patterns (data not shown). RA alone did not exert any significant activity towards global DNA methylation modulation, whereas the augmentation in global DNA methylation % was evident upon treatment with Bel (global DNA methylation increased by 15–38% after 6–24 hrs treatment with 0.2 μM Bel, whereas in later time-points the previous methylation level was restored). In contrast, the increase in DNA methylation level upon combined treatment Bel + RA was more sudden and more pronounced compared with treatment with Bel alone (DNA methylation increased by 40% after 6 hrs treatment). It should be highlighted that prolonged treatment with Bel + RA (72 hrs) down-regulated the global DNA methylation more than 14%.

### Treatment with belinostat induces increased association of acetylated histone H4 with p27 promoter

**Figure 5:**
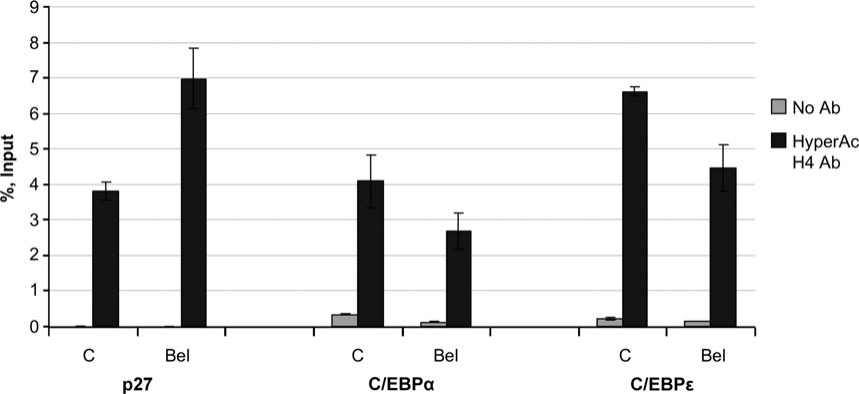
Bel effect on acetylated histone H4 association with p27, C/EBPα and C/EBPε promoter regions. ChIP with antibody against hyperacetylated histone H4 was performed with control (C) and NB4 cells treated with 2 μM Bel for 6 hrs (Bel). Specimens were further tested using qPCR analysis. Data are represented as percent input (± SD, *n* = 2).

To examine, if the increase in p27 gene expression after treatment with Bel can be attributed to or correlated with histone H4 hyperacetylation, ChIP analysis was performed. Indeed, upon NB4 cells treatment with 2 μM Bel for 6 hrs, histone H4 in p27 promoter region was almost twice more hyperacetylated compared with untreated cells (Fig.5). This suggests, that histone H4 hyperacetylation may be one of the factors leading to increased p27 mRNA level.

It is widely admitted, that transcription factors C/enhancer binding protein alpha (EBPα) and C/EBPε play a crucial role in granulocytic differentiation 31,32. Our group previously demonstrated 22 that 24 hrs NB4 cells treatment with Bel induced C/EBPα mRNA expression twofold, whereas later C/EBPα mRNA level decreased. In addition, only low effect on C/EBPε mRNA expression was detected after treatment with Bel as a single agent. Combined treatment of 0.2 μM Bel + 1 μM RA has also demonstrated to be less effective in C/EBPε mRNA level up-regulation compared with RA alone, despite the evidence that Bel in combination with RA enhances and accelerates RA-induced granulocytic differentiation.

In this study we investigated, whether Bel treatment has an effect on histone H4 hyperacetylation level at C/EBPα and C/EBPε promoter regions. However, in accordance to gene expression data, no increase in H4 hyperacetylation at C/EBPα and C/EBPε promoter regions was detected, which also coincided with NBT test data, demonstrating that Bel alone is unable to induce granulocytic differentiation.

### Belinostat modulates protein complexes associated with hyperacetylated histone H4

We were interested, if the increase in basal histone H4 hyperacetylation level after NB4 cells treatment with Bel is accompanied by compositional changes in protein complexes that are found in association with this epigenetic mark. Therefore, we used Co-IP and subsequent MS analysis that helped us to reveal proteins that are associated with hyperacetylated histone H4 in control and Bel-treated NB4 cells. Quantitative changes of identified proteins in control and Bel-treated cells are presented in Table2 as C/Bel ratio (the mark ‘C’ denotes that protein was detected only in control cells, whereas mark ‘Bel’ indicates that protein was seen only in treated cells).

**Table 2:** Summary of identified NB4 cells proteins found in complexes with hyperacetylated histone H4 in control and Bel-treated cells

No.	Accession	Gene name	Score	C/Bel ratio*	Function
1	Q5QNW6	HIST1H2AH	9505.53	0.77105	Core component of nucleosome
2	Q99878	HIST1H2AJ	8305.59	0.59452	Core component of nucleosome
3	P33778	HIST1H2BB	42,815.45	1	Core component of nucleosome
4	P58876	HIST1H2BD	10,401.13	0.51171	Core component of nucleosome
5	P57053	HIST2H2BF	45,639.36	Bel	Core component of nucleosome
6	P84243	H3F3A	10,974.41	1.10517	Core component of nucleosome
7	Q6NXT2	H3F3C	828.6	0.69768	Core component of nucleosome
8	P62805	HIST1H4A	17,505.7	0.84366	Core component of nucleosome
9	P16401	HIST1H1B	615.33	1.1853	Nucleosomal condensation
10	P16403	HIST1H1C	2448.72	1.05127	Nucleosomal condensation
11	Q71UI9	H2AFV	5543.68	1.23368	Replaces conventional H2A in a subset of nucleosomes
12	P57053	H2BFS	10,401.13	0.5886	Replaces conventional H2A in a subset of nucleosomes
13	P0C0S5	H2AFZ	7646.1	Bel	Replaces conventional H2A in a subset of nucleosomes
14	Q14181	POLA2	152.92	C	DNA replication
15	Q9BZD3	GCOM1	224.06	C	Component of Pol II(G) complex
16	P0CAP2	POLR2M	284.39	C	Component of Pol II(G) complex
17	P18615	NELFE	266.18	C	Represses RNA polymerase II transcript elongation
18	P51504	ZNF80	444.63	Bel	Transcriptional regulation
19	P18124	RPL7	235.63	C	Translation apparatus regulation
20	P47914	RPL29	622.4	0.92312	Translation apparatus regulation
21	Q9BWG6	SCNM1	620.47	C	RNA splicing
22	Q8WXA9	SREK1	148.46	Bel	Regulation of alternative splicing
23	P19338	NCL	132.66	C	Pre-rRNA transcription and ribosome assembly
24	P02788	LTF	275.73	C	Antimicrobial and anti-inflammatory activity
25	P61626	LYZ	751.9	3.56085	Bacteriolysis
26	P06702	S100A9	2362.67	Bel	Antimicrobial activity. Phagocyte migration promotion. Apoptosis
27	P05109	S100A8	1869.7	Bel	Antimicrobial activity. Phagocyte migration promotion. Apoptosis
28	P60709	ACTB	3806.68	1.82212	Cell motility
29	P63261	ACTG1	1713.96	0.34301	Cell motility
30	Q562R1	ACTBL2	664.65	Bel	Cell motility
31	A6NHL2	TUBAL3	110.36	C	Microtubule element
32	Q71U36	TUBA1A	452.48	C	Microtubule element
33	P07437	TUBB	620.75	2.2034	Microtubule element
34	Q9BQS8	FYCO1	61.54	C	May mediate microtubule plus end-directed vesicle transport
35	Q13326	SGCG	315.83	C	Component of sarcoglycan complex
36	Q9NY65	TUBA8	123.57	Bel	Microtubule element
37	Q9BQE3	TUBA1C	54.26	Bel	Microtubule element
38	O15144	ARPC2	666.79	Bel	Regulation of actin polimerization
39	Q96A32	MYLPF	737.84	Bel	Myosin light chain
40	Q6UY14	ADAMTSL4	247.27	C	Positive regulation of apoptosis
41	P47929	LGALS7	392.7	Bel	Apoptosis regulation. Pro-apoptotic
42	Q08378	GOLGA3	7.44	Bel	Golgi str. maintenance. Cleavage product necessary for apoptotic response
43	P50897	PPT1	124.76	Bel	Lysosomal degradation. DNA fragmentation during apoptosis
44	Q5M775	SPECC1	380.22	C	Proto-oncogene
45	P25054	APC	30.36	Bel	Tumour suppressor
46	Q04760	GLO1	253.31	C	Involved in the regulation of TNF-induced transcriptional activity of NF-κB
47	Q5T200	ZC3H13	22.03	C	Down-regulation of NF-κB pathway
48	O95989	NUDT3	215.22	C	Signal transduction. Negatively regulates ERK1/2 pathway
49	Q99665	IL12RB2	269.83	C	Signalling component coupling to the JAK2/STAT4 pathway. Promotes the proliferation of T-cells as well as NK cells
50	Q8IV04	TBC1D10C	834.75	0.8781	Ras signalling pathway inhibition
51	Q96NH3	C6orf170	1777.55	C	Controls ciliary morphology. Involved in Hedgehog signal transduction
52	P06748	NPM1	612.64	1.85893	Regulates tumour suppressors TP53/p53 and ARF. Chaperone
53	Q9NNW7	TXNRD2	158.36	Bel	Implication in the defences against oxidative stress
54	P29762	CRABP1	133.35	Bel	Regulates access of retinoic acid to the nuclear retinoic acid receptors
55	P17066	HSPA6	121.58	Bel	Chaperone
56	P48741	HSPA7	78.01	Bel	Chaperone
57	P11142	HSPA8	144.69	Bel	Chaperone. Repressor of transcriptional activation
58	P55735	SEC13	122	C	May be involved in protein transport
59	P62987	UBA52	755.27	0.77105	Proteosomal degradation, chromatin structure maintenance, gene expression regulation and stress response
60	P0CG47	UBB	241.72	C	Proteosomal degradation, chromatin structure maintenance, gene expression regulation and stress response
61	Q6ZMR5	TMPRSS11A	860.74	Bel	Probable serine protease
62	P00738	HP	1190.67	Bel	Makes haemoglobin accessible to degradative enzymes
63	Q6S8J3	POTEE	346.61	C	Protein and ATP binding
64	A5A3E0	POTEF	369.2	2.13828	Protein and ATP binding
65	P0CG39	POTEJ	107.66	1.46228	Protein and ATP binding
66	Q9BTF0	THUMPD2	238.14	C	RNA binding. Methyltransferase activity
67	Q68CQ7	GLT8D1	206.26	C	Glycosyltransferase
68	A6NIV6	LRRIQ4	145.7	Bel	Leucine-rich repeats and IQ motif containing

* ‘C’ denotes that protein is seen in control only. ‘Bel’ – detected in treated cells only.

**Figure 6:**
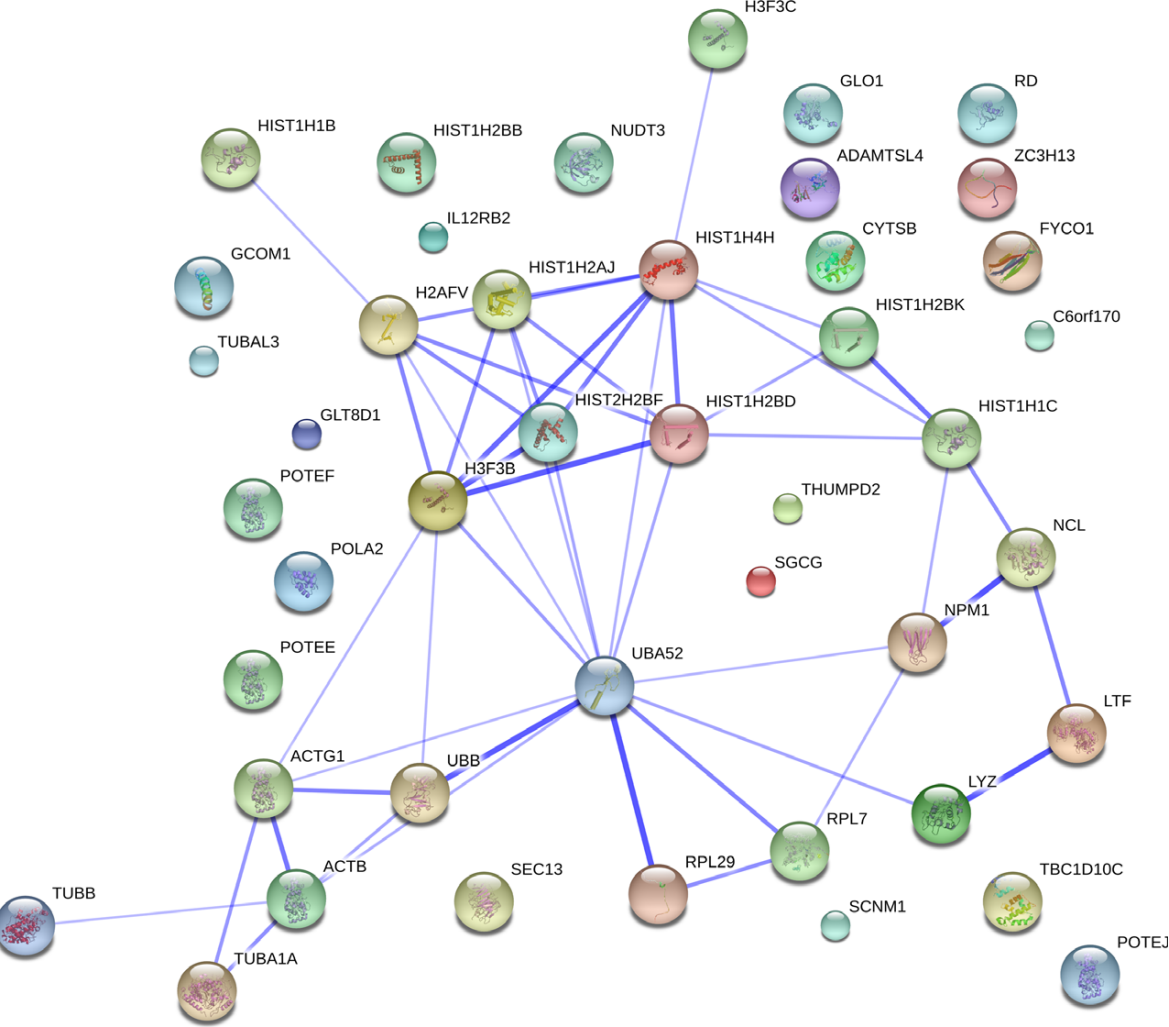
Proteins identified in association with hyperacetylated histone H4 in control NB4 cells. Untreated NB4 cells were subjected to ChIP - MS analysis. Association network of identified proteins was studied and represented using STRING database (http://string.embl.de).

In untreated NB4 cells hyperacetylated histone H4 was found to associate with 45 different proteins (Table2). The network of proteins associated with hyperacetylated histone H4 in control NB4 cells is presented in Figure6. In control cells only, hyperacetylated histone H4 was found associated with proteins that are involved in DNA replication (POLA2), transcription (GCOM1, POLR2M, NELFE, NCL), translation (RPL7) and RNA splicing (SCNM1). Also hyperacetylated histone H4 was identified, associated with proto-oncogene SPECC1, regulator of apoptosis ADAMTSL4, as well as with proteins involved in different signalling cascades: NF-κB, JAK2/STAT4, Ras and Hedgehog signal transduction pathways. Interestingly, hyperacetylated histone H4 in control NB4 cells was found to be associated with Nucleophosmin (NPM), a protein which regulates tumour suppressors TP53/p53 and ARF and is shown to be overexpressed in actively proliferating cells, such as various cancer and stem cells 33. It is worth mentioning that NPM was found in complexes with hyperacetylated histone H4 after treatment with 2 μM Bel as well, but to a much lesser extent.

**Figure 7:**
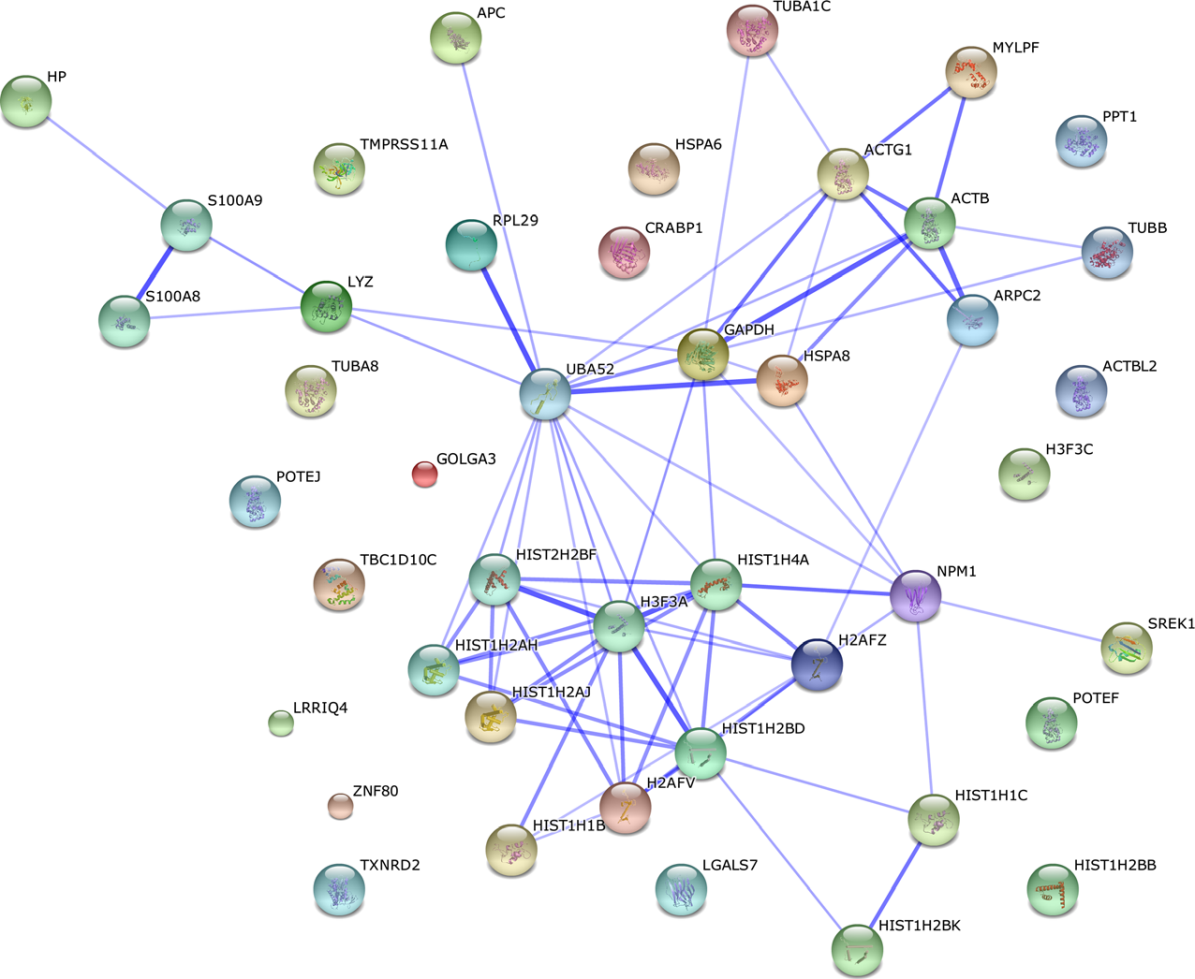
Proteins identified in association with hyperacetylated histone H4 in Bel treated NB4 cells. 2 μM Bel treated NB4 cells were subjected to ChIP - MS analysis. Association network of identified proteins was studied and represented using STRING database (http://string.embl.de).

After 6 hrs treatment with 2 μM Bel (Table2, Fig.7) hyperacetylated histone H4 was identified to be associated with proteins that are pro-apoptotic and necessary for apoptotic response (S100A9, S100A8, LGALS7, GOLGA3, PPT1). Tumour suppressor APC was found in immunoprecipitated complexes as well. It is important to underline that hyperacetylated histone H4 has been also found to be associated with proteins that are involved in the defence against oxidative stress (TXNRD2) and access of RA to the nuclear retinoic acid receptors regulation (CRABP1).

## Discussion

It is widely accepted that epigenetic modifiers and changes in the epigenetic landscape play a very significant role in APL pathogenesis. The onset of this haematological malignancy, at least in part, was shown to be induced by abnormal HDACs recruitment to RA target genes 3,34. Therefore, activation of the RA signalling pathway *via* HDAC activity inhibition may serve as a promising strategy in APL differentiation therapy.

In this study, we investigated the effect of a novel HDACi belinostat, alone or in combination with differentiation inducer RA, on human promyelocytic leukaemia NB4 and HL-60 cells growth and granulocytic differentiation. Notably, belinostat in combination with RA indeed showed a valuable activity towards APL cells. Combined treatment of 0.2 μM Bel + 1 μM RA inhibited NB4 and HL-60 cells proliferation and arrested cell cycle in G_0_/G_1_ phase. In addition, combined treatment was shown to accelerate and induce higher percent of granulocytic differentiation, compared to treatment with RA alone. These results are similar to the effect of some other well-known HDACi inhibitors used for leukaemia granulocytic differentiation therapy. Our group previously demonstrated a comparable effect on NB4 and HL-60 cells granulocytic differentiation upon combined treatment with HDACi Phenyl butyrate or BML-210 with RA 35,36. Also it was demonstrated that HDACi FK228 in HL-60 cells increases RA-induced granulocytic differentiation more than 1.8-fold, whereas this combination has only a minor pro-differentiational effect on NB4 cells 37.

Considering molecular mechanisms, associated with APL cell growth suppression and increased granulocytic differentiation in more detail, the effect of Bel, RA and Bel + RA treatments on NB4 cells certain epigenetic modifiers (HDAC1, HDAC2, PCAF) and cell cycle regulators (p27) gene and protein expression were evaluated. Our group have showed numerous times earlier 22,23 that although NB4 and HL-60 cells differ in the way that NB4 is a typical APL cell line, possessing t(15;17), whereas HL-60 does not have PML-RARα, the differences in their responses to various HDACi treatments are minimal. The manner of the effect is similar or identical, only kinetics can sometimes be found to be different. Therefore, further investigations were restricted to NB4 cells only.

We showed, that treatment with belinostat sharply down-regulated NB4 cells HDAC1 and HDAC2 mRNA levels. Reduction in HDAC1 protein level was also observed, together with an increase in histone H4 hyperacetylation. In addition, our group previously demonstrated 22 that belinostat induces accumulation of H4K16Ac mark, which in turn is associated with the transcriptional activation. It was also shown that belinostat up-regulates H3K9 acetylation level depending on treatment duration and used dose 22.

As it has been described earlier 38–40, in AML cells HDACs, predominantly HDAC1, are overexpressed, which leads to histone hypoacetylation, whereas during granulocytic differentiation HDAC1 protein expression diminishes and acetylation patterns are restored. Therefore, the results we observed in NB4 cells using belinostat alone and in combination with RA are promising and in line with published data.

We also were interested in belinostat’s effect not only on HDACs but on HATs as well, in this case particularly on PCAF. This protein has shown to function as an acetylase, as it directly modifies histones and other proteins. Furthermore, it associates with additional activators, such as CBP/p300, ACTR 41 and is able to increase histone acetylation at transcriptional sites, targeting predominantly histones H3 and H4 42. It has been demonstrated earlier 43 that RA induces PCAF expression and accumulation in P19 carcinoma cells’ nucleus. It also has been postulated that increase in PCAF mRNA levels potentiates retinoid-dependent gene expression 44. In accordance to this data, we showed that in NB4 cells, RA indeed induced PCAF gene expression. However, in this study we also revealed that 6 hrs exposure with belinostat reduced PCAF gene expression more than twofold, in comparison with control cells, whereas later PCAF gene expression was restored. Furthermore, in a previous research 22 the dose-dependent down-regulation in PCAF protein level upon treatment with belinostat has been demonstrated. Therefore, it seems that belinostat, indeed, perturbs histone acetylating enzymes machinery in APL cells, but the precise mechanism still remains elusive and needs further investigation. It can only be speculated on the basis of Hirano *et al*. research 45, that down-regulation of PCAF gene and protein expression may be associated with belinostat’s pro-apoptotic effect, as it was demonstrated that down-regulation of PCAF sensitized human prostate cancer PC3 cells to chemotherapeutic therapy, induced G_1_ arrest and apoptosis.

Because of HDACs association with histone methyl transferases and DNA methyl transferases (DNMTs) belinostat exerts an impact not only on HDACs or HATs but on other chromatin remodelling enzymes as well. Our previous research 22 demonstrated that treatment with Bel depletes Polycomb repressive complex 2 subunits’ EZH2 and SUZ12 proteins, however, not bearing any significant effect on H3K27 trimethylation. In addition, in this study the activity of Bel and combined treatment Bel + RA on global DNA methylation level was also evaluated. Upon 72 hrs treatment with Bel + RA global NB4 cells DNA methylation level was down-regulated more than 14%. This is in agreement with Arzenani *et al*. 46, who showed that HDACi Trichostatin A down-regulates DNMT1 gene and protein expression and reduces global DNA methylation in the hepatoma cells.

In addition, our group previously indicated 22 that, although belinostat promotes APL granulocytic differentiation, as a single agent it has no effect on NB4 cells C/EBPα and C/EBPε genes expression or genes coding for transcription factors that drive granulocytic differentiation 47,48. In this study, using ChIP, we aimed to determine histone H4 hyperacetylation level in C/EBPα and C/EBPε promoter regions. We found that upon treatment with belinostat alone, basal histone H4 hyperacetylation level was up-regulated, but no increase in histone H4 hyperacetylation levels in C/EBPα and C/EBPε promoter regions were detected. Therefore, this suggests that other mechanisms than direct activation of C/EBPα and C/EBPε are responsible for ability of belinostat to enhance RA-induced granulocytic differentiation. We suppose that belinostat may mainly accomplish its action *via* effects on the cell cycle. RT-qPCR analysis revealed that upon NB4 cells treatment with 0.2 μM belinostat, p27 gene expression is firmly up-regulated (more than fourfold after 72 hrs treatment). In addition, ChIP results indicated the obvious increase in hyperacetylated histone H4 association with p27 promoter region. It was demonstrated earlier 22, that belinostat up-regulates NB4 cells p27 protein levels as well. Taken this data together, it is plausible not to underestimate the role of p27 activation in belinostat-mediated antileukaemic effect.

Very interesting results, regarding belinostat’s activity on APL cells, were revealed by MS analysis. We found that upon 6 hrs treatment with 2 μM belinostat hyperacetylated histone H4 was no longer associated with proteins involved in gene transcription or translation, as it was the case in untreated NB4 cells, but was found to associate with proteins, that are usually detected in cytosolic fraction as components of neutrophil extracellular traps (NETs). To express in more detail, NETs were shown to be a composition of DNA, histones and antimicrobial proteins, that form an extracellular mesh able to trap and kill pathogens 49 and they are released during a cell death that depends on reactive oxygen species (ROS) produced by the NADPH oxidase complex 50.

Regarding composition of NETs, it has been indicated that NETs contain calprotectin (S100A8/S100A9) 51 a protein complex composed of two calcium-binding proteins which are abundantly found in neutrophils cytosolic fraction and have shown to have apoptosis inducing activity 52. Upon NB4 cells treatment with belinostat MS analysis results indeed revealed the association of hyperacetylted histone H4 with calprotectin (both S100A8 and S100A9). Calprotectin is essential for the neutrophilic NADPH oxidase activation 53. The importance of S100A8/A9 complex in NB4 cells NADPH oxidase activation has been demonstrated by the S100A9 expression blockage, after which NADPH oxidase activity has been impaired 54. In addition, S100A8/A9-dependent NADPH stimulation has shown to increase ROS levels in keratinocytes 55. Notably, in PANC-1 cells belinostat promoted ROS production 56. It has been shown for multiple myeloma cells as well 57.

Furthermore, besides the calprotectin, we also found a probable serine protease TMPRSS11A associated with hyperacetylated histone H4, which is in agreement with data, showing that NETs contain serine proteases, as they may execute antimicrobial functions in those structures 58. Taking all together, we assume that belinostat’s cell death-inducing activity in some manner may relate to NETs formation. Although, it is already known that belinostat triggers apoptosis in myeloid cells 22,20,21, not NETosis (the different mechanism of cell death when NETs are released, named by Steinberg and Grinstein 59), the possibility that belinostat intervenes in NETs formation may not be rejected completely.

It is evident, that APL cells, despite of their differentiation state, manage to generate very few NETs, which was demonstrated for HL-60 cells 60. This might be explained by their inability to induce autophagy, as not only NADPH activity, but also autophagy has shown to be crucial for NETosis 61. In addition, recently it was demonstrated, that NETs formation does not necessarily require cell death 62,63 and that cells, which have formed NETs, may retain the capacity to die *via* apoptosis 64 However, possible role of belinostat in triggering NETs formation in APL cells is only an educated guess, at least at this stage. Therefore, further investigations are needed to confirm or reject this hypothesis.

Summarizing, our findings, regarding belinostat’s effect on cell growth, differentiation, gene and protein expression, as well as on epigenetic modifications, confirmed potential value belinostat has in APL therapy. In this study some new insights in possible molecular mechanisms of belinostat were also revealed.
